# Integrated genomic analysis of biological gene sets with applications in lung cancer prognosis

**DOI:** 10.1186/s12859-017-1737-2

**Published:** 2017-07-11

**Authors:** Su Hee Chu, Yen-Tsung Huang

**Affiliations:** 1 Department of Epidemiology, School of Public Health, Brown University, 121 S Main St, Providence, RI USA; 2 Department of Biostatistics, School of Public Health, Brown University, 121 S Main St, Providence, RI USA; 3 Institute of Statistical Science, Academia Sinica, No. 128, Section 2, Academia Rd, Taipei City, Taiwan; 4 Channing Division of Network Medicine, Brigham and Women’s Hospital Harvard Medical School, 181 Longwood Ave, Boston, MA USA

**Keywords:** Pathway analysis, Data integration, Epigenetics, Gene expression, Gene set analysis, Integrative genomics

## Abstract

**Background:**

Burgeoning interest in integrative analyses has produced a rise in studies which incorporate data from multiple genomic platforms. Literature for conducting formal hypothesis testing on an integrative gene set level is considerably sparse. This paper is biologically motivated by our interest in the joint effects of epigenetic methylation loci and their associated mRNA gene expressions on lung cancer survival status.

**Results:**

We provide an efficient screening approach across multiplatform genomic data on the level of biologically related sets of genes, and our methods are applicable to various disease models regardless whether the underlying true model is known (iTEGS) or unknown (iNOTE). Our proposed testing procedure dominated two competing methods. Using our methods, we identified a total of 28 gene sets with significant joint epigenomic and transcriptomic effects on one-year lung cancer survival.

**Conclusions:**

We propose efficient variance component-based testing procedures to facilitate the joint testing of multiplatform genomic data across an entire gene set. The testing procedure for the gene set is self-contained, and can easily be extended to include more or different genetic platforms. iTEGS and iNOTE implemented in R are freely available through the inote package at https://cran.r-project.org//.

**Electronic supplementary material:**

The online version of this article (doi:10.1186/s12859-017-1737-2) contains supplementary material, which is available to authorized users.

## Background

Burgeoning interest in integrative analyses has produced a rise in studies which incorporate data from multiple genomic platforms. In general, there are two methods of integrating genomic data [1]. The first is horizontal integration, where genomic data from different studies but of the same type (e.g. multiple gene- expression microarray studies) are combined, sometimes across labs, cohorts, and platforms. The second is vertical integration, where multiple levels of ’omics data (e.g. DNA variation, methylation, and gene expression) are gathered on the same subjects and analyzed. A useful distinction to be made in methods for vertical integrative approaches involves whether the multiplatform data are assessed via a “screen-and-clean” paradigm [2, 3], where each platform is analyzed independently to screen for and select a subset of significant candidates to use in a combined analysis (i.e. a sequential integration analysis), or whether the multiplatform data are assessed simultaneously (i.e. a joint integration analysis).

Most integrative studies employ approaches that primarily rely on dimension reduction methods to accommodate the high dimensionality of analyzing multiple platforms [4, 5]. These techniques seek to synthesize complex genetic information into summary statistics, potentially at the cost of discarding large quantities of data which might still be mechanistically informative. And while methods development for non-reductive multi-platform integrative analysis has become more common in recent years [6, 7], these methods are mainly restricted to candidate gene interrogations, and do not encapsulate the highly likely network-level interactions between disease-risk-conferring genes. Of course, numerous tests of gene sets are available [8–10] – but few that also include the integration of additional genomic platforms.

Additionally, literature for conducting formal hypothesis testing on an integrative gene set level is considerably more sparse than that for estimation. For example, integrative methods for identifying potential risk pathways include strategies that employ Bayesian mixture modeling [11–14], Bayesian graphical models [13], Bayesian network models [15], non-negative matrix factorization [16–18], and weighted gene correlation network approaches [3]. To our knowledge, methods for joint integrative testing of any kind are small in number; for gene sets, there is a variant of GSEA [4, 5], and for candidate gene approaches there are a few multivariate and mediation methods [6, 7, 19]. Although effect estimation is informative when candidate gene sets/networks are already identified or hypotheses are well-defined, an efficient screening approach across multi-platform genomic data is critical for hypothesis generation. Therefore, in this paper, we focus on efficient testing procedures to assess the effect of an entire gene set through the joint analysis of multiple genomic platforms, such as epigenomic and transcriptomic data.

**Fig. 1:**
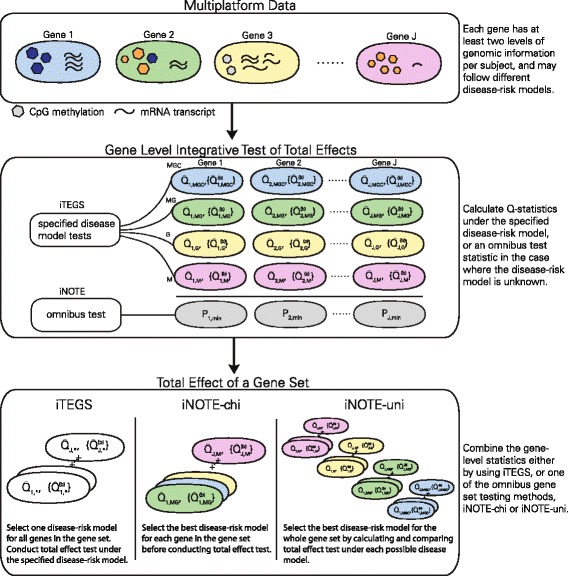
A general overview of the variance component-based total effect gene set testing procedure. Each gene within a gene set of interest has at least two sources of genomic data such as DNAm and mRNA expression per subject. Two levels of integration occur, first at the single-gene level to jointly test DNAm and mRNA expression, then at the network level where the evidence from all viable genes is jointly assessed to produce a test of the gene set. *Q* ^_∗_: observed Q-statistics; $\{\hat {Q}_{*}^{(b)}\}$: the resampling-based perturbation distribution for $\hat {Q}_{*}$ under the null

Joint integrative analyses become substantially challenging when considered on the level of gene sets, where the number of model parameters rapidly increases as the size of the gene set grows. Additionally, correlation structure within a gene on the level of methylation sites, as well as between genes on the transcript expression level, may cause conventional univariate or multivariate tests to perform poorly [10, 20, 21]. We therefore propose a variance component test to assess the total effect of a set of methylation loci and mRNA gene expressions across a gene set on disease outcome. The test statistic for the joint gene set analysis follows a mixture of *χ* ^2^ distributions, which we may approximate analytically, or empirically using a perturbation procedure, after specifying a disease model for the whole gene set (e.g. epigenetic effect only, or epigenetic effect and gene expression effect, or both epigenetic and gene expression effect as well as their interactions). However, because the true disease models underlying different genes may vary, we also construct two gene set level omnibus tests to accommodate different disease models. A general overview of our approach is presented in Fig. 1.

The biological motivation for this paper lies in the connection between DNA methylation (DNAm) patterns and lung cancer survival. In particular, we are interested in the total joint effect of DNAm and downstream mRNA expression levels for all genes in a related pathway on survival probability in 559 subjects with both epigenome-wide DNAm and RNA-sequencing data from The Cancer Genome Atlas (TCGA). We demonstrate the utility of our integrative testing procedures by identifying significant gene sets that can be further explored for potential biomarkers of prognosis or even therapeutic targets.

## Methods

Our integrative gene set testing approach can be viewed as a variance component test [6, 10] under the generalized linear mixed model framework [22].

### Integrated gene model and test of total effects

Huang et al. [6] proposed a method to jointly analyze the effects of a set of genetic markers and a corresponding measure of gene expression within a single candidate gene on disease outcome, which is applicable to the analysis of epigenetic and transcriptomic data. Briefly, let *Y*_*i*_ represent the dichotomous disease outcome of subject *i* (*i*=1,…,*n*) and let ***X***_*i*_ represent *r* covariates of interest for subject *i*. Further assume that *Y*_*i*_ is associated with the *r* covariates of interest ***X***_*i*_ (with the first covariate set as the intercept), the methylation levels at a set of *p* CpG loci within the candidate gene (***M***_*i*_=(*M*_1*i*_,…,*M*_*pi*_)^′^), the corresponding gene expression (*G*_*i*_), and possibly their interactions. Then, the underlying model for any given candidate-gene total effect test is:

1 $$ \begin{aligned} \text{logit}\left\{\mathbb{P}\left(Y_{i}\right.\right.&\left.\left.=1~|~\boldsymbol{M}_{i},G_{i},\boldsymbol{X}_{i}\right)\right\} = \boldsymbol{X}_{i}^{\prime}\boldsymbol{\beta}_{X}\\ &\quad+\boldsymbol{M}_{i}^{\prime}\boldsymbol{\beta}_{M}+G_{i}\beta_{G}+G_{i}\boldsymbol{M}_{i}^{\prime}\boldsymbol{\beta}_{C}, \end{aligned}  $$

where $\boldsymbol {\beta }_{X}=\left (\beta _{X_{1}},{\ldots },\beta _{X_{r}}\right)^{\prime }$, $\boldsymbol {\beta }_{\boldsymbol {M}}=\left (\beta _{M_{1}},{\ldots },\beta _{M_{p}}\right)^{\prime }$, *β*_*G*_, $\boldsymbol {\beta }_{C}=\left (\beta _{C_{1}},{\ldots },\beta _{C_{p}}\right)^{\prime }$ represent the regression coefficients for the covariates, the CpG loci, gene expression, and the interactions between the CpG set and gene expression, respectively. Then, the null hypothesis for a single-gene test of total effect is:

2 $$ H_{0}:\boldsymbol{\beta}_{M}=\boldsymbol{0},\quad\beta_{G}=0,\quad\boldsymbol{\beta}_{C}=\boldsymbol{0},  $$

which can be cast into a variance component testing framework by assuming: 1) the elements of ***β***_*M*_ are independent and follow an arbitrary distribution with mean 0 and variance *τ*_*M*_ and 2) the elements of *β*_*C*_ are independent and follow an arbitrary distribution with mean 0 and variance *τ*_*C*_. In other words, the outcome model (1) becomes a logistic mixed model and the null hypothesis may be re-expressed as:

3 $$ H_{0}: \tau_{M}=\tau_{C}=0,\quad\beta_{G}=0.  $$

Using the above model specifications, the score statistics may be derived for *τ*_*M*_,*β*_*G*_ and *τ*_*C*_ respectively as:

$$\begin{array}{*{20}l} U_{\tau_{M}}&=\left(\boldsymbol{Y}-\Hat{\boldsymbol{\mu}}_{0}\right)^{\prime}\mathbb{MM}^{\prime}\left(\boldsymbol{Y}-\Hat{\boldsymbol{\mu}}_{0}\right),\\ U_{\beta_{G}}&=\boldsymbol{G}^{\prime}\left(\boldsymbol{Y}-\Hat{\boldsymbol{\mu}}_{0}\right),\\ U_{\tau_{C}}&=\left(\boldsymbol{Y}-\Hat{\boldsymbol{\mu}}_{0}\right)^{\prime}\mathbb{CC}^{\prime}\left(\boldsymbol{Y}-\Hat{\boldsymbol{\mu}}_{0}\right), \end{array} $$

where $\mathbb {M}=\left (\boldsymbol {M}_{1},{\ldots },\boldsymbol {M}_{n}\right)^{\prime }$, ***G***=(*G*_1_,…,*G*_*n*_)^′^, $\mathbb {C}=\left (\boldsymbol {C}_{1},{\ldots },\boldsymbol {C}_{n}\right)^{\prime }$, ***C***_*i*_=*G*_*i*_ ***M***_*i*_, ***μ*^**_0_=(*μ̂*_01_,…,*μ̂*_0*n*_)^′^, and $\Hat {\mu }_{0i}=e^{X_{i}^{\prime }\Hat {\boldsymbol {\beta }_{X}}}/\left (1+e^{X_{i}^{\prime }\Hat {\boldsymbol {\beta }_{X}}}\right)$ is the mean *Y*_*i*_ under the null model

4 $$ \text{logit}\left\{\mathbb{P}\left(Y_{i}=1~|~\boldsymbol{M}_{i},G_{i},\boldsymbol{X}_{i}\right)\right\}=\boldsymbol{X}_{i}^{\prime}\boldsymbol{\beta}_{X}  $$

where ***β***_*X*_ ^ is the maximum likelihood estimator of ***β***_*X*_. Using a conventional approach to combine the score statistics for each component such that $Q_{conv}=\boldsymbol {U}^{\prime }\mathcal {I}^{-1}\boldsymbol {U}$, where $\boldsymbol {U}=(U_{\tau _{M}}, U_{\beta _{G}}, U_{\tau _{C}})$), would involve combining score statistics from different scales and requires the existence of the 8th moment of ***Y*** to calculate the efficient information matrix of ***U***, $\mathcal {I}$. Therefore, the component score statistics are instead summed to create a weighted test statistic for the null hypothesis (3), denoted as *Q*_∗_ statistics:

$$\begin{array}{*{20}l} \begin{aligned} Q_{MGC}&=n^{-1}\left(a_{1}U_{\tau_{M}}+a_{2}U_{\beta_{G}}^{2}+a_{3}U_{\tau_{C}}\right).\\ Q_{MG} &=n^{-1}\left(a_{1}U_{\tau_{M}}+a_{2}U_{\beta_{G}}^{2}\right),\\ Q_{M} &=n^{-1}\left(a_{1}U_{\tau_{M}}\right),\\ Q_{G} &=n^{-1}\left(a_{2}U_{\tau_{\beta_{G}}}^{2}\right), \end{aligned} \end{array} $$

where *Q*_∗_={*Q*_*MGC*_,*Q*_*MG*_,*Q*_*M*_,*Q*_*G*_} represents the underlying disease models MGC, MG, M, and G which correspond to the model specifications that include 1) CpG, gene expression, and their interactions across the full gene set, 2) the CpG and gene expression effects across the full gene set, 3) only CpG effect, and 4) only gene expression effect respectively, and the weights *a*_1_, *a*_2_, and *a*_3_ defined as the inverse square root of the variances for their corresponding score statistics to make $U_{\tau _{M}}$, $U_{\beta _{G}}^{2}$ and $U_{\tau _{C}}$ comparable.

Because $U_{\tau _{M}}$, $U_{\beta _{G}}^{2}$, and $U_{\tau _{C}}$ are all quadratic functions of ***Y***, the null distribution of *Q*_∗_ may be approximated with a mixture of *χ* ^2^ distributions, thus we may derive *p*-values for *Q*_∗_ by using the Satterthwaite scaled- *χ* ^2^ approximation [23] or the characteristic function inversion method [24]. Alternatively, one can perform the test by conducting a resampling-based perturbation procedure [25–27]. The perturbation procedure is used to approximate the null distribution of *Q*=*Q*(***β***_*X*_ ^) by resampling realizations of its asymptotic distribution under *H*_0_. Specifically, it can be shown that

$$\begin{array}{*{20}l} Q_{*}\rightarrow\sum_{l}\left(\boldsymbol{A}_{l}^{\prime}\boldsymbol{\epsilon}\right)^{2}, \end{array} $$

where ***ε*** is a multivariate normal random variable with mean 0 and covariance $\boldsymbol {D}=\left (\begin {array}{cc} \boldsymbol {D}_{XX} & \boldsymbol {D}_{XV} \\ \boldsymbol {D}_{VX} & \boldsymbol {D}_{VV} \end {array}\right)=n^{-1}\boldsymbol {U}^{\prime }\boldsymbol {W}\boldsymbol {U}$, ***U***=(***U***_1_,…,***U***_*n*_)^′^, $\boldsymbol {U}_{i}=(\boldsymbol {X}_{i}^{\prime },\boldsymbol {V}_{i}^{\prime })$, $\boldsymbol {V}_{i}=(\sqrt {a_{1}}\boldsymbol {M}_{i}^{\prime }, \sqrt {a_{2}}G_{i},\sqrt {a_{3}}\boldsymbol {C}_{i}^{\prime })^{\prime }$, ***W***=diag{*μ*_0*i*_(1−*μ*_0*i*_)}, and ***A***_*l*_ is the *l*th row of $\boldsymbol {A}=\left [-\boldsymbol {D}_{XV}^{\prime }\boldsymbol {D}_{XX}^{-1},\boldsymbol {\mathrm {I}}_{2p+1}\right ]$ where ***I*** is the (2*p*+1) dimensional identity matrix. In other words, *Q*_∗_ can be shown to follow a mixture of *χ* ^2^ distributions. The perturbation procedure then approximates the asymptotic distribution of *Q*_∗_ by generating realizations of ***ε***, ***ε̂***, repeatedly, where $\hat {\boldsymbol {\epsilon }}=n^{-1/2}\sum _{i=1}^{n}\boldsymbol {U}_{i}^{\prime }(Y_{i}-\hat {\mu }_{0i})\mathcal {N}_{i}$ and $\mathcal {N}_{i}$ are independent *N*(0,1). For perturbation *b*, we generate $\mathcal {N}^{(b)}=\left (\mathcal {N}_{1}^{(b)},{\ldots },\mathcal {N}_{n}^{(b)}\right)$, *b*=1,…,*B* (the number of perturbations) to obtain the realization of the distribution of ***ε***, from which we approximate the distribution of *Q*_∗_.

### Integrated gene set model and test of total effects

We expand our model to extend the single-gene joint test proposed by Huang et al. [6] to a full gene set. Let *J*×1 vector ***G***_*i*_ represent the expression level for *j*=1,…,*J* genes for subject *i*, and $\boldsymbol {M}_{i}=\left (\boldsymbol {M}_{1i}^{\prime },{\ldots },\boldsymbol {M}_{ji}^{\prime },{\ldots },\boldsymbol {M}_{Ji}^{\prime }\right)^{\prime }$, represent the *K*×1 methylation value vector for the *p*_*j*_ CpG loci of gene *j* with $\boldsymbol {M}_{ji}=\left (M_{1i},{\ldots },M_{p_{j}i}\right)^{\prime }$, $K=\sum _{j}p_{j}$. Similarly, to allow for interaction effects, let $\boldsymbol {C}_{i}=\left (\boldsymbol {C}_{1i}^{\prime },{\ldots },\boldsymbol {C}_{ji}^{\prime },{\ldots },\boldsymbol {C}_{Ji}^{\prime }\right)^{\prime }$, where $\boldsymbol {C}_{ji}=\left (G_{ji}M_{1i},{\ldots },G_{ji}M_{p_{j}i}\right)^{\prime }$. The model thus underlying a *gene set* test which includes interactions between the methylation sites and gene expression can be specified as:

5 $$ \begin{aligned} \text{logit}\left\{\mathbb{P}\left(Y_{i}\right.\right.&\left.\left.=1~|~\boldsymbol{M}_{i},\boldsymbol{G}_{i},\boldsymbol{X}_{i}\right)\right\} = \boldsymbol{X}_{i}^{\prime}\boldsymbol{\beta}_{X}\\ &\quad+\boldsymbol{M}_{i}^{\prime}\boldsymbol{\beta}_{M}+\boldsymbol{G}_{i}^{\prime}\beta_{G}+\boldsymbol{C}_{i}^{\prime}\boldsymbol{\beta}_{C}, \end{aligned}  $$

where $\boldsymbol {\beta }_{M}=\left (\boldsymbol {\beta }_{M_{1}}^{\prime },{\ldots },\boldsymbol {\beta }_{M_{J}}^{\prime }\right)^{\prime }_{K\times 1}$, $\boldsymbol {\beta }_{G}=\left (\beta _{G_{1}},\beta _{G_{2}},{\ldots },\beta _{G_{J}}\right)^{\prime }_{J\times 1}$, and $\boldsymbol {\beta }_{C}=\left (\boldsymbol {\beta }_{C_{1}}^{\prime },{\ldots },\boldsymbol {\beta }_{C_{J}}^{\prime }\right)^{\prime }_{K\times 1}$ represent the coefficients for all CpG loci, gene expression, and within-gene cross-product interactions across the gene set, and $\boldsymbol {\beta }_{M_{j}}=\left (\beta _{M_{j1}},{\ldots },\beta _{M_{jp_{j}}}\right)^{\prime }_{p_{j}\times 1}$ and $\boldsymbol {\beta }_{C_{j}}=\left (\beta _{C_{j1}},{\ldots },\beta _{C_{jp_{j}}}\right)^{\prime }_{p_{j}\times 1}$. The resulting hypothesis test for the total effect of a gene set is:

6 $$ \begin{aligned} H_{0}:\boldsymbol{\beta}_{M}=\boldsymbol{0},\quad\boldsymbol{\beta}_{G}=\boldsymbol{0},\quad\boldsymbol{\beta}_{C}=\boldsymbol{0}. \end{aligned}  $$

As the gene set grows, however, the number of parameters to test becomes intractable under standard likelihood-based multivariate testing methods. Similar to the above single gene analyses, we resort to an empirical Bayes approach by assuming that the effect parameters *β*’s share common distributions for each gene *j*: 1) the elements of $\boldsymbol {\beta }_{M_{j}}$ are independent and follow an arbitrary distribution with mean 0 and variance $\tau _{M_{j}}$ and 2) the elements of $\boldsymbol {\beta }_{C_{j}}$ are independent and follow another arbitrary distribution with mean 0 and variance $\tau _{C_{j}}$. Based on the above assumptions, we construct a test for the following null hypothesis:

7 $$ \begin{aligned} H_{0}:\tau_{M_{j}}=\tau_{C_{j}}=0,\quad\beta_{Gj}=0,\quad\text{for } j=1,{\ldots}J. \end{aligned}  $$

We use a modified variance component testing procedure to obtain our test statistic, *Q*_Net∗_. For the gene set being tested:

8 $$ \begin{aligned} Q_{\text{Net}*}&=\sum_{j=1}^{J}w_{j}Q_{j}=n^{-1}\left(\boldsymbol{Y}-\hat{\boldsymbol{\mu}}_{0}\right)^{\prime}\\ &\quad\times\left(w_{1}K_{1*}+\cdots+w_{J}K_{J*}\right)\left(\boldsymbol{Y}-\Hat{\boldsymbol{\mu}}_{0}\right), \end{aligned}  $$

where *K*_*j*∗_ indicates the kernel of the underlying disease model specification for gene *j*: $K_{j*}=a_{1j}\mathbb {M}_{j}\mathbb {M}_{j}^{\prime }+a_{2j}\boldsymbol {G}_{j}\boldsymbol {G}_{j}^{\prime }+a_{3j}\mathbb {C}_{j}\mathbb {C}_{j}^{\prime }$ for the MGC model, and $K_{j*}=a_{1j}\mathbb {M}_{j}\mathbb {M}_{j}^{\prime }+a_{2j}\boldsymbol {G}_{j}\boldsymbol {G}_{j}^{\prime }$, $K_{j*}=a_{1j}\mathbb {M}_{j}\mathbb {M}_{j}^{\prime }$, and $K_{j*}=a_{2j}\boldsymbol {G}_{j}\boldsymbol {G}_{j}^{\prime }$ for the MG, M, and G only models, respectively; we again chose the weights *w*_1_,…,*w*_*J*_ to be the inverse of the standard deviation to make each *Q*_*j*_ comparable. In closed form calculations, we assume all genes follow the same model specification: M, G, MG, or MGC such that we obtain as test statistics *Q*_Net*M*_, *Q*_Net*G*_, *Q*_Net*MG*_, or *Q*_Net*MGC*_. We note that the disease-model specifying only gene expression effects is in fact equivalent to the single-platform (i.e. non-integrative) gene set testing method proposed by Huang and Lin [10] with working independence among the genes. Their approach, called the total effect of a gene set (TEGS), is therefore a special case of the integrative methods presented here.

Under the null, *Q*_Net∗_ can be shown to follow a mixture of *χ* ^2^ distributions. Thus, as in the single-gene total effect test, we may calculate *p*-values for *Q*_Net∗_ either by using the characteristic function inversion method (Davies method), the resampling-based perturbation procedure, or approximate by matching the first two moments of the scaled- *χ* ^2^ distribution (Satterthwaite method). We will refer to this method as the *integrated* total effect of a gene set (iTEGS) with iTEGS-M, iTEGS-G, iTEGS-MG and iTEGS-MGC denoting tests under the M, G, MG, and MGC models, respectively.

### Integrated pathway-wide omnibus tests

#### Omnibus chi-squared gene set test

A gene set drawn from a network or pathway is comprised of many genes, and each of these genes may have different underlying disease models wherein causal relationships with disease risk might be best represented by differing models M, G, MG, and MGC. The algorithm to obtain the empirical null distribution of the sum of *χ* ^2^ statistics of the gene set is as follows:

1. For each gene *j* in the gene set:
  - Calculate the observed *Q* ^_*jM*_, then obtain its empirical distribution $\left \{\Hat {Q}_{jM}^{(b)}, b=1,{\ldots },B\right \}$ where *B* denotes the number of perturbations.
  - Repeat a.) for *Q* ^_*jG*_, *Q* ^_*jMG*_, and *Q* ^_*jMGC*_ respectively.
  - Obtain *p*-values $\text {Pr}\left (\Hat {Q}_{j*}^{(b)}>Q_{j*}\right)$ for *Q* ^_*jM*_, *Q* ^_*jG*_, *Q* ^_*jMG*_, *Q* ^_*jMGC*_. Denote these as *P* ^_*jM*_,*P* ^_*jG*_,*P* ^_*jMG*_, and *P* ^_*jMGC*_, respectively, and $\Hat {P}_{j_{\min }}=\min \left (\Hat {P}_{jM}, \Hat {P}_{jG}, \Hat {P}_{jMG}, \Hat {P}_{jMGC}\right)$. Transform $\Hat {P}_{j_{\min }}$ to its corresponding $\chi ^{2}_{1}$ quantile denoted $\Hat {T}_{j_{\min }}$ (the $\chi ^{2}_{1}$ statistic with tail probability $\Hat {P}_{j_{\min }}$).
  - Obtain the empirical distribution of $\Hat {T}_{j_{\min }}$, $\left \{\Hat {T}_{j_{\min }}^{(b)}\right \}$ where $\Hat {T}_{j_{\min }}^{(b)}$ is the *χ* ^2^ statistic with tail probability of $\Hat {P}_{j_{\min }}^{(b)}=\min \left (\Hat {P}_{jM}^{(b)},\Hat {P}_{jG}^{(b)},\Hat {P}_{jMG}^{(b)},\Hat {P}_{jMGC}^{(b)}\right)$
2. Sum the *J* observed $\Hat {T}_{j_{\min }}$ across the gene set such that $\Hat {T}_{\text {Net}}=\sum _{j=1}^{J}\Hat {T}_{j_{\min }}$. To obtain the empirical null for *T* ^_Net_, calculate $\left \{\Hat {T}_{\text {Net}}^{(b)}=\sum _{j=1}^{J}\Hat {T}_{j_{\min }}^{(b)}\right \}$. Calculate the gene-set p-value by obtaining the proportion of values that are more extreme than the observed *T* ^_Net_.

This approach, which we term the chi-transformed integrated network omnibus total effect test (iNOTE-chi), should provide a powerful approach for testing gene sets in cases where the true underlying disease models for the genes in a gene set are unknown.

#### Omnibus uniform network model gene set test

While iNOTE-chi provides the flexibility that different genes may follow different disease models (M, G, MG or MGC), its performance may depend on whether the true underlying models for each gene are correctly selected, which introduces another source of uncertainty in model specification. In cases where the disease risk signal is not easily differentiable between the disease risk models, omnibus selection of disease models for each gene may not necessarily improve the power of the method. Therefore, we developed another test that determines a consensus disease model that is most generally applicable across the whole gene set. The complete algorithm is as follows:

1. For each gene *j* in the gene set:
  - Calculate the observed *Q* ^_*jM*_, then obtain its empirical distribution $\left \{\Hat {Q}_{jM}^{(b)}, b=1,{\ldots },B\right \}$ where *B* denotes the number of perturbations.
  - Repeat a.) for *Q* ^_*jG*_, *Q* ^_*jMG*_, and *Q* ^_*jMGC*_ respectively.
2. Sum the *J* observed *Q* ^_*j*∗_ across the gene set under each disease model such that we have three test statistics: *Q* ^_Net*M*_, *Q* ^_Net*G*_, *Q* ^_Net*MG*_, *Q* ^_Net*MGC*_. Calculate their associated *p*-values $\text {Pr}\left (\Hat {Q}_{\text {Net}*}^{(b)}>\Hat {Q}_{\text {Net}*}\right)$, denoted *P* ^_Net∗_, then select as our omnibus network test statistic:
  $$\Hat{P}_{\text{Net}_{\min}}=\min\left(\Hat{P}_{\text{Net}M},\Hat{P}_{\text{Net}G}, \Hat{P}_{\text{Net}MG},\Hat{P}_{\text{Net}MGC}\right) $$
3. Obtain the empirical null for $\Hat {P}_{\text {Net}_{\min }}$ by calculating
$\left \{\Hat {P}_{\text {Net}_{\min }}^{(b)} =\min \left (\Hat {P}_{\text {Net}M}^{(b)}, \Hat {P}_{\text {Net}G}^{(b)}, \Hat {P}_{\text {Net}MG}^{(b)},\Hat {P}_{\text {Net}MGC}^{(b)}\right)\right \}$. Calculate the gene set *p*-value as above by comparing the observed $\Hat {P}_{\text {Net}_{\min }}$ to $\left \{\Hat {P}_{\text {Net}_{\min }}^{(b)}\right \}$ and obtaining the proportion of values that are more extreme than the observed $\Hat {P}_{\text {Net}_{\min }}$, or by using the Satterthwaite method.

We term this approach the uniform model integrated network omnibus total effect test (iNOTE-uni).

### Simulation studies

We simulated DNAm based on Infinium HumanMethylation 450K Beadchip data obtained from the lung tissue samples of 681 lung cancer patients in The Cancer Genome Atlas. To realistically simulate disease outcome and gene expression, high correlation CpG blocks were identified across the epigenome to generate CpG sets which were then used to model gene expression. One causal CpG was selected per CpG set and gene expression was simulated for each subject *i* by the linear regression model: $\phantom {\dot {i}\!}\boldsymbol {G}_{i}=\delta _{0}+\boldsymbol {M}_{j_{\text {causal}}}\delta +\boldsymbol {\epsilon }_{i}$, where $\boldsymbol {\epsilon }_{i}\thicksim \mathcal {MVN}(\boldsymbol {0}, \boldsymbol {\Sigma })$ and ***Σ*** is a *J*×*J* covariance matrix with diag(1) and between-gene covariance equal to 0.7. Within-gene covariance was accounted for by the covariance structure in actual subject data (from which the CpG blocks were drawn). For each simulation, a case-control sample of 100 cases and 100 controls were randomly selected from a simulated cohort of 681 subjects.

To evaluate the performance of the proposed omnibus methods, iNOTE-chi and iNOTE-uni, we conducted power simulations for gene set sizes of 10 and 50 at signal density proportions (i.e. the proportion of genes randomly selected to be causal within the gene sets) of 0.2, 0.5, 0.8, 1.0 across seven different simulation settings. The seven scenarios varied the mixture of underlying disease models for the causal genes in a given gene set as follows: 1) all genes follow M-only models; 2) all genes follow MG models; 3) all genes follow MGC models; 4) 50:50 mixture of M-only and MG models; 5) 50:50 mixture of M-only and MGC models; 6) 50:50 mixture of MG and MGC models; 7) one-third mixture of M, MG, MGC models.

We next compared our proposed methods, iTEGS, iNOTE-chi, and iNOTE-uni with two existing methods: 1) gene set association analysis (GSAA) [5], an integrative variant of the common gene set enrichment analysis (GSEA) approach to gene set testing, and 2) a more recent estimating equation-based integrative method proposed by Zhao et al. [7] which assumes that any effects of the exposure (e.g., methylation) are fully mediated by a mediator (e.g., gene expression) to produce the outcome which we will simply refer to as the ‘Zhao’ method. The Zhao method requires estimation of parameters and thus struggles to converge if the size of the gene set gets too large (e.g., the number of genes is greater than 5). To accommodate the competing method, we reduced the size of the gene set to three genes, each with 11 corresponding CpG loci, but note that the number of parameters is still quite large (i.e., 36 main effect parameters) relative to our sample size. To compare the power performance of GSAA which tests for a competitive null hypothesis [28], 49 background gene sets of equal size (3 genes per set) and null effect on disease risk were simulated in the same manner as the causal gene set in each simulation.

### Application: pathway-wide association scans in TCGA

To illustrate the utility of our method, we obtained an initial sample of pre-processed level 3 genomic data from 681 lung adenocarcinoma (LUAD) and lung squamous cell carcinoma (LUSC) patients in The Cancer Genome Atlas (TCGA) database (http://cancergenome.nih.gov/) with DNAm data assayed on the Illumina Infinium Human Methylation 450K. Among the 681 subjects, 559 also had measured mRNA expression and clinical outcome data. From the 559 patients with both levels of genomic data, we identified a final analytic sample of 249 subjects who had complete information on one-year survival since cancer diagnosis. Methylation and RNA-Seq data were adjusted for batch effects using the ComBat method in the Surrogate Variable Analysis (sva) Bioconductor package [29].

To obtain candidate pathways to test, we next scanned the Molecular Signatures Database (MsigDB; version 5.1) [4] for all gene sets that were associated with the keywords “lung” and “(cancer OR carcinomas)” in *homo sapiens*, and identified 103 gene sets of varying sizes (ranging from as small as 5 to as large as 456 genes in the gene set) for joint testing with integration of epigenomic and transcriptomic data. Among these, four gene sets were excluded due to the absence of methylation probes, mRNA expression data, or both, in all the genes that comprised each gene set, resulting in a final 99 gene sets for our joint analyses. The 99 gene sets were then scanned using iTEGS under the M, MG, and MGC disease-risk models, as well as with the two iNOTE methods. The iTEGS-G test, assuming mRNA gene expression effects only, was calculated to provide a benchmark for assessing the benefits of integrating methylation data, and incorporated in the iNOTE omnibus model selection algorithm. Finally, all gene set tests were adjusted for potential confounding covariates: smoking history (pack years), sex, age at diagnosis, race (white, black, other), pathologic tumor stage at time of initial biopsy, and cell type (adenocarcinoma, squamous cell carcinoma).

## Results

### Simulation study

#### Size and power

With the gene set size of 50, type I errors were protected for the variance component test statistics of iTEGS under each of the three gene set models assuming all causal genes within the set follow M, MG, or MGC models (Table 1). The iNOTE-uni method was also well protected with a type I error rate close to 0.05. The type I error rate of iNOTE-chi was 0.052 under the gene set size of 10 but slightly inflated when the gene set became larger: 0.067 for the gene set size of 25 and 0.08 for the gene set size of 50.

**Table 1:** Empirical sizes of the proposed variant-component based tests

	Davies	Perturbation
iTEGS-M	0.043	0.041
iTEGS-MG	0.048	0.048
iTEGS-MGC	0.045	0.045
iNOTE-chi	-	0.085
iNOTE-uni	-	0.046

Type I error was calculated for a gene set of size 50 using 5000 simulations and significance threshold of *α*=0.05

**Fig. 2:**
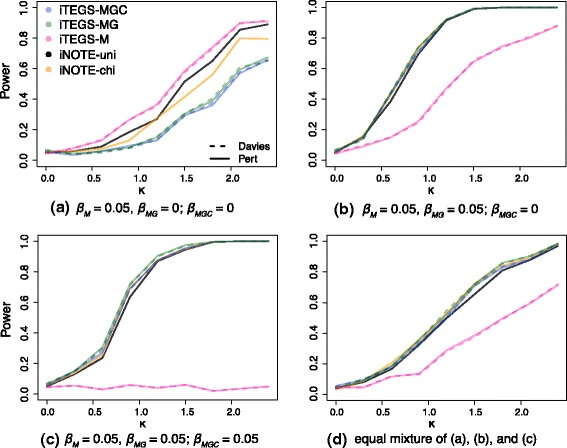
Internal power simulations across various disease-model settings for moderately sized gene sets. Power performance is shown for a gene set of size 50 with a 20% causal risk signal proportion of genes under the disease model settings where all causal genes contribute to disease via **a** methylation effect only (M); **b** methylation and mRNA expression effect (MG); **c** methylation, mRNA expression, and their interactive effects (MGC); **d** equal mixtures of M, MG, and MGC. *κ* on the x-axis denotes the coefficient multiplier for each of the effects *β*_*M*_, *β*_*MG*_, and *β*_*MGC*_

To evaluate the performance of the iNOTE methods with respect to power, we conducted power simulations for a set of 50 genes with signal density of 20% (i.e. 10 genes with one causal CpG locus). Power curves for simulation settings where all causal genes follow 1) M, 2) MG, 3) MGC, and 4) an approximately equal mixture of M, MG, and MGC disease-risk models are presented in Fig. 2. Other mixtures of disease risk models were also assessed but results were similar to those of the fourth simulation setting (Additional file 1: Figure A.1). Increasing the causal signal density proportion from 20% to 80% resulted in sharp increases in power across all simulation settings, as expected (Additional file 1: Figure A.2).

In the first simulation setting where all 10 causal genes in the gene set follow the M disease-risk model, iTEGS-M demonstrates the greatest power, as expected (Fig. 2 a). The other two model formulations, iTEGS-MG and iTEGS-MGC, over-specify gene expression and interaction parameters for testing and thus suffer a performance loss in power. Similarly, in the simulation setting under the MG model, iTEGS-MG, which correctly specifies the model, has the most optimal power performance, with iTEGS-MGC achieving very similar power performance (Fig. 2 b). However, iTEGS-M performs considerably worse under settings where both methylation and gene expression effects are present. In the third simulation setting where the methylation-by-expression interaction terms are present (i.e., the MGC model) and the true disease risk model is MGC, iTEGS-MGC and iTEGS-MG again have similar power performance, but iTEGS-M demonstrates a steep drop in power as it tests only for the presence of a portion of the signal (Fig. 2 c). The final simulation setting in which the causal genes are randomly assigned to M, MG, or MGC disease-risk models in equal proportion, the performance between the different iTEGS statistics is similar to the second simulation setting (Fig. 2 d).

Notably, across all simulation settings, the iNOTE-chi and iNOTE-uni tests reveal strong power performance that is nearly equivalent to the iTEGS under the correctly specified model, with the exception of the first simulation setting, where they are slightly less powerful. In the first simulation setting, iNOTE-uni outperforms iNOTE-chi; but in all other simulation settings however, iNOTE-chi exhibits a slight power advantage compared to iNOTE-uni, particularly in the case of mixtures of different causal-disease-risk models across different causal genes within a given gene set.

#### Comparison to existing approaches

**Fig. 3:**
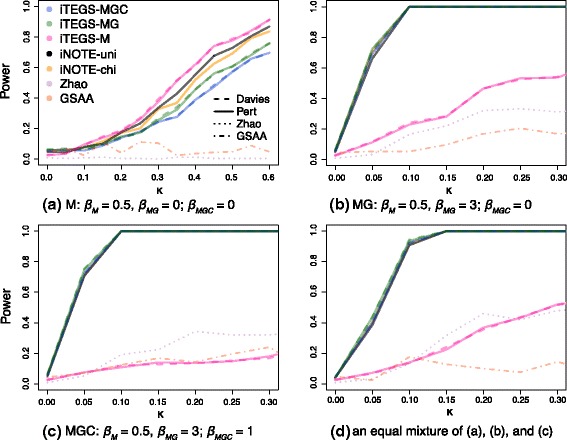
Power simulations comparing variance-component score-based gene set testing procedures to existing methods. Power performance is shown for a gene set of 3 causal genes with a 100% causal risk signal proportion under the disease model settings where all causal genes contribute to disease via **a** methylation effect only (M); **b** methylation and mRNA expression effect (MG); **c** methylation, mRNA expression, and their interactive effects (MGC); **d** equal mixtures of M, MG, and MGC. *κ* on the x-axis denotes the coefficient multiplier for each of the effects *β*_*M*_, *β*_*MG*_, and *β*_*MGC*_

We also studied the performance of iTEGS and the two iNOTE tests in comparison to two competing approaches to integrative analysis, GSAA and the Zhao method using the same four simulation settings described in the internal power comparisons (to review power performance for additional mixtures of disease-risk models, see Additional file 1: Figure B.1) In the 3-gene setting, our methods behave as in the 50-gene simulations where the correctly specified iTEGS demonstrates optimal power performance. Importantly, both omnibus approaches, iNOTE-uni and iNOTE-chi, and the correctly specified iTEGS tests consistently outperform GSAA and the Zhao method under various simulation settings (Fig. 3). Our variance component-based tests especially dominate the Zhao method in the presence of high direct CpG methylation effects and strong correlation between methylation loci and gene expression (Fig. 3 a), which suffers from major power loss due to the presence of only direct methylation effects, rather than mediated effects through gene expression. The power of the Zhao method is somewhat recovered in simulation settings where the gene expression signal exists. The GSAA method, which tests for a competitive null hypothesis, achieved very low power across all of the simulation settings.

### Application: lung cancer survival associated gene sets

We next analyzed the TCGA lung cancer data using iTEGS (under each of the M-only, MG and MGC models), iNOTE-chi, and iNOTE-uni. Among the 99 lung cancer associated MsigDB gene sets that were tested, iTEGS identified 57, 59, and 52 significant gene sets (p<0.05) under the MGC, MG, and M model specifications, and iNOTE-chi and iNOTE-uni identified 51 and 58 significant gene sets respectively (Table 2). The counts of identified gene sets using our proposed methods all exceeded what we expected under the null, i.e., 5. Gene sets that were identified as significantly associated with one-year survival after Bonferroni correction at p<5×10^−4^ in at least one of each of the iTEGS and iNOTE tests are reported in Table 3. The *p*-values obtained with the Davies method for the iTEGS statistics were generally quite similar to the perturbation-based empirical *p*-values when the gene set sizes were small, but tended to vary when the gene sets grew in size (Additional file 1: Table C.1).

**Table 2:** Counts of overlapping significant lung cancer gene sets associated with one-year survival by iTEGS, iNOTE, and GSAA

		iTEGS					iNOTE			GSAA
		MGC	MG	M	G		chi	uni		
	MGC	57 (27)	55 (25)	41 (13)	40 (13)		49 (20)	53 (25)		5 (1)
	MG		59 (27)	44 (15)	39 (12)		50 (20)	54 (27)		6 (1)
	M			52 (17)	27 (4)		38 (10)	46 (16)		5 (0)
iTEGS	G				40 (13)		37 (11)	39 (12)		3 (0)
	chi						51 (23)	48 (20)		5 (1)
iNOTE	uni							58 (28)		6 (1)
GSAA										8 (1)

A total of 99 lung cancer associated gene sets were obtained and tested from MsigDB. Tests for iTEGS were calculated under disease-risk model specifications M: methylation effect only, G: gene expression effect only, MG: methyation and mRNA expression effects, and MGC: methylation effect, mRNA expression effect, and their interactions. The total and overlapping counts of significant gene sets identified by each method is reported here, with numbers in parentheses denoting the counts of gene sets that remain significant after Bonferroni correction at p<5×10^−4^

**Table 3:** Variance component-based total effect test *p*-values for gene sets associated with lung cancer after Bonferroni correction

			Approximated *P*-Values				Empirical *P*-Values				Omnibus *P*-Values		
	N_0_	N _*T*_	Q _MGC_	Q _MG_	Q _M_	Q _G_	Q _MGC_	Q _MG_	Q _M_	Q _G_	iNOTE _chi_	iNOTE _uni_	GSAA
BRUECKNER TARGETS OF MIRLET7A3 DN	78	71	1.1E-05	1.7E-06	5.5E-04	2.4E-04	<1E-04	<1E-04	8E-04	3E-04	8E-04	<1E-04	0.325
BRUECKNER TARGETS OF MIRLET7A3 UP	111	106	1.3E-06	4.1E-07	2.5E-04	1.3E-04	<1E-04	<1E-04	4E-04	1E-04	2E-04	<1E-04	0.230
COLDREN GEFITINIB RESISTANCE DN	230	216	8.0E-09	3.2E-10	1.2E-09	8.9E-04	<1E-04	<1E-04	<1E-04	9E-04	<1E-04	<1E-04	0.095
DAUER STAT3 TARGETS UP	49	49	1.6E-04	4.6E-05	0.002	0.003	7E-04	2E-04	0.003	0.002	4E-04	3E-04	<1E-04
DCA UP.V1 DN	193	163	6.1E-06	4.9E-06	0.003	1.3E-04	<1E-04	<1E-04	0.004	<1E-04	<1E-04	<1E-04	0.920
DCA UP.V1 UP	191	162	7.3E-05	4.7E-05	0.002	0.003	1E-04	1E-04	0.002	0.004	<1E-04	<1E-04	0.065
HALMOS CEBPA TARGETS DN	46	44	2.4E-04	9.5E-05	0.018	5.4E-04	8E-04	<1E-04	0.021	4E-04	0.001	<1E-04	0.645
HATADA METHYLATED IN LUNG CANCER UP	390	356	9.3E-07	2.6E-07	2.8E-06	0.003	<1E-04	<1E-04	<1E-04	0.003	<1E-04	<1E-04	0.900
KIM MYC AMPLIFICATION TARGETS UP	201	169	2.2E-06	1.3E-06	0.004	2.9E-05	<1E-04	<1E-04	0.005	<1E-04	<1E-04	<1E-04	0.605
KOBAYASHI EGFR SIGNALING 24HR DN	251	228	7.3E-04	1.7E-05	9.6E-07	0.109	9E-04	<1E-04	<1E-04	0.110	0.015	<1E-04	0.920
KOBAYASHI EGFR SIGNALING 24HR UP	101	91	1.7E-09	1.9E-08	9.0E-04	3.2E-06	<1E-04	<1E-04	0.001	<1E-04	<1E-04	<1E-04	0.035
KOBAYASHI EGFR SIGNALING 6HR DN	18	18	5.4E-05	9.5E-06	2.5E-04	0.003	<1E-04	<1E-04	9E-04	0.004	<1E-04	<1E-04	0.110
KRAS.600.LUNG.BREAST UP.V1 DN	289	261	3.4E-04	3.4E-04	0.020	0.003	7E-04	2E-04	0.023	0.004	<1E-04	4E-04	0.125
KRAS.600.LUNG.BREAST UP.V1 UP	288	247	8.8E-07	2.3E-06	0.100	7.9E-07	<1E-04	<1E-04	0.105	<1E-04	<1E-04	<1E-04	0.060
KRAS.AMP.LUNG UP.V1 UP	144	121	9.0E-05	1.7E-04	0.288	1.6E-05	5E-04	4E-04	0.284	1E-04	<1E-04	<1E-04	0.045
KRAS.DF.V1 UP	193	175	3.3E-08	1.4E-08	3.1E-04	5.1E-06	<1E-04	<1E-04	4E-04	<1E-04	1E-04	<1E-04	0.160
KRAS.LUNG UP.V1 UP	141	126	5.3E-10	5.1E-09	0.025	1.1E-08	<1E-04	<1E-04	0.026	<1E-04	<1E-04	<1E-04	0.575
LI AMPLIFIED IN LUNG CANCER	178	165	1.6E-04	6.1E-05	9.5E-05	0.031	3E-04	1E-04	1E-04	0.032	0.009	<1E-04	0.180
LOCKWOOD AMPLIFIED IN LUNG CANCER	214	205	1.6E-04	6.7E-06	6.7E-07	0.081	2E-04	<1E-04	<1E-04	0.081	0.014	<1E-04	0.230
SHEDDEN LUNG CANCER GOOD SURVIVAL A12	317	269	7.9E-10	9.7E-09	0.003	1.4E-07	<1E-04	<1E-04	0.002	<1E-04	<1E-04	<1E-04	0.385
SHEDDEN LUNG CANCER GOOD SURVIVAL A4	196	186	6.0E-06	5.7E-06	5.7E-05	0.006	<1E-04	<1E-04	2E-04	0.007	<1E-04	<1E-04	0.470
SHEDDEN LUNG CANCER POOR SURVIVAL A6	456	411	2.2E-08	1.4E-11	8.1E-11	7.6E-04	<1E-04	<1E-04	<1E-04	0.001	1E-04	<1E-04	0.900
SWEET KRAS ONCOGENIC SIGNATURE	89	81	0.010	3.8E-04	3.1E-05	0.157	0.011	4E-04	<1E-04	0.158	0.019	<1E-04	0.180
SWEET KRAS TARGETS DN	66	59	3.1E-08	2.3E-09	5.0E-04	6.5E-07	<1E-04	<1E-04	0.001	<1E-04	<1E-04	<1E-04	0.655
TBK1.DF DN	287	266	2.9E-08	3.9E-09	1.3E-05	2.3E-05	<1E-04	<1E-04	<1E-04	<1E-04	5E-04	<1E-04	0.570
TBK1.DF UP	290	275	0.006	0.001	2.4E-04	0.174	0.008	0.002	2E-04	0.172	0.051	2E-04	0.265
TOOKER GEMCITABINE RESISTANCE DN	122	115	4.1E-05	1.1E-05	8.8E-05	0.006	<1E-04	<1E-04	2E-04	0.007	0.004	<1E-04	0.710
ZHONG RESPONSE TO AZACITIDINE AND TSA UP	183	158	1.1E-05	9.8E-07	3.0E-07	0.024	1E-04	<1E-04	<1E-04	0.025	1E-04	<1E-04	0.935

Satterthwaite-approximated and empirical *p*-values for all significant gene sets after Bonferroni correction by at least one of the iTEGS and at least one of the iNOTE tests. Empirical *p*-values and approximated *p*-values are very similar, irrespective of the sizes of the gene sets tested. N_0_: total no. of genes in the gene set; N _*T*_: total no. of genes with methylation and gene expression data available (i.e. tested); Q: the iTEGS Q-statistic test specifying M, G, MG, or MGC; Bonferroni adjusted *p*-value threshold was calculated as *α*/*M*=5×10^−04^, where *α*=0.05 and M is the total number of gene sets tested

A total of 28 gene sets were identified as significant by at least one of the iTEGS tests and by at least one of the omnibus iNOTE tests. There were 23 and 28 gene sets with significant iNOTE-chi and iNOTE-uni *p*-values after Bonferroni correction, respectively. Interestingly, the iTEGS-MGC, iTEGS-MG, iNOTE-chi and iNOTE-uni outperformed the iTEGS-G in their ability to identify gene sets significantly associated with one-year survival which were known a priori to be related to lung cancer, despite the fact that many of the gene sets curated by the MsigDB were obtained from gene expression studies. This is supportive of the notion that screening of gene sets using efficiently integrated multiplatform ‘omic data can increase the ability to identify potentially mechanistic disease pathways. Similar patterns supporting the utility of integrative analysis also emerged in additional exploratory gene set screening analyses with different outcomes (e.g. pathological stage of tumor at initial biopsy) and in different pathway databases (e.g. BIOCARTA and KEGG pathways, which include gene sets not specific to lung cancer) can be viewed in Additional file 1: Tables D.1-D.3, E.1, and E.2.

The GSAA method only identified 8 significant gene sets, of which only one survived a Bonferroni adjustment. This is a predictable feature of the adapted Kolmogorov-Smirnov algorithm employed by the GSAA approach, which ignores between-gene correlation among the genes in a gene set and instead uses relative gene rankings among all possible genes under consideration. Thus, the GSAA approach is dependent on not only the size of the gene set being tested, but also the proportion of significantly associated genes belonging to a gene set of interest versus the proportion that does not. Indeed, GSAA may not reliably retrieve disease-associated gene sets when the proportion of signal genes in the gene set is small, even if the associations are strong and highly significant.

**Fig. 4:**
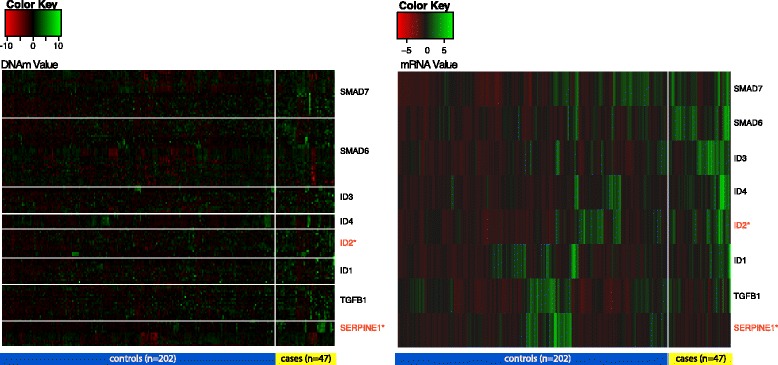
CpG methylation (left) and mRNA expression (right) for the Gautschi SRC signaling pathway. Two heatmaps are shown separated on the x-axis by one-year survival status, which represents non-survivors and survivors at one year post initial diagnosis, respectively, and on the y-axis by individual genes within the gene set. Gene names highlighted in red were significant at p<0.01

Among the top gene sets identified by iTEGS and iNOTE in Table 3, we recovered several involving KRAS expression and EGFR signaling, both of which are canonical genes implicated in cancer literature, as well as others related to a microRNA associated with cancer, mir-let7a3. We also retrieved several gene sets previously identified as predictive of lung cancer survival, lending further credibility to both the integrative approach and our findings. For illustrative purposes, we created methylation and mRNA expression heatmaps for one small but interesting gene set which was identified as associated with one-year survival in our analyses: the Gautschi SRC signaling gene set (*p*-values: iTEGS-MGC=0.017, iTEGS-MG=0.030, iTEGS-M=0.653; iTEGS-G=0.007; iNOTE-chi=0.005, iNOTE-uni=0.015; GSAA=0.205) [30], which is comprised of a set of highly down-regulated genes in lung cancer cell lines after the application of an SRC inhibitor. Refined characterization of the individual genes viable for testing in the gene set showed that non-survivors had generally higher mRNA expression values than survivors (Fig. 4); these findings are biologically consistent with those of Gautschi et al. [30] that SRC inhibition, and therefore reduced expression of genes in the Id family, is associated with decreased cancer cell invasion.

## Discussion

Our proposed approach has two advantages: first, it is a variance component-based score test where the testing procedure is constructed under the null without estimating the large number of effect parameters; second, the omnibus tests approach the optimal performance demonstrated under correct model specification by synthesizing the evidence from three candidate models and are thus robust to model misspecification. In our simulation studies, we found that iTEGS and iNOTE dominated two competing methods, GSAA and the Zhao method. All three tests use information across multiple genomic platforms. However, the GSAA first discards information by using weighted *p*-values across individual genes to integrate different genomic data, and then performs an adapted Kolmogorov-Smirnov test which assesses a competitive null hypothesis [28]. The Zhao method requires strong assumptions that all methylation effects on disease risk are mediated through gene expression, and struggles to converge when the ratio of parameters to the sample size is too large or when there is strong correlation between CpG loci. Although our simulations assumed causal associations between DNAm and gene expression, our testing procedures remain legitimate tests of joint effect even in cases where such associations do not exist. It should also be noted that the original development of GSAA and the Zhao method had slightly different purposes than the multiplatform integrated analyses of a gene set. For example, GSAA focuses on examining signal enrichment within a gene set by testing competitive hypotheses rather than self-contained null hypotheses; the Zhao method was designed to gain power by exploiting eQTL (expression quantitative trait loci) effects. Their suboptimal performance demonstrates the imperative need for an efficient screening test specifically intended for the joint analysis of gene sets by integration of multiplatform genomic data.

The perturbation procedures used in iNOTE-chi and iNOTE-uni are the main source of computational burden in the omnibus approaches; however, it is worth noting that perturbation procedures resample from the asymptotic null distributions of the gene-level Q statistics, and thus both 1) preserve the covariance structure within and between genes when conducting gene set tests and 2) are far more efficient than permutation procedures requiring direct reshuffling of the data. It is additionally much easier to adjust for covariates using perturbation procedures than using permutations particularly when there exist associations between the genetic data and the covariates. To run one simulation in the MG-only setting using a 2.60GHz Intel Xeon E5-2670 CPU to test 50 genes with 11 CpG sites and 1000 perturbations each, the approximate computation time is 29, 31, and 32 seconds for iTEGS (any model specification), iNOTE-chi, and iNOTE-uni respectively. For the Davies approximation to iTEGS, the computation time is about 22, 22, and 25 seconds for the M, MG, and MGC model specifications respectively.

In our data application to the lung cancer survival data, we were able to recover a sizeable number of significant gene sets. Many of these gene sets tended to be least significant when tested under the iTEGS statistic with only the DNAm disease-risk model specification, but grew increasingly more significant with the inclusion of mRNA gene expression and interaction specifications. This is biologically plausible in that given a true gene pathway, it is highly unlikely that the CpG sites that biologically map within the bounds of a given gene will behave in a strictly linear manner; the remainder of the significant signal in these gene sets can be deduced to arise from the synergistic or antagonistic interaction effects between DNAm and mRNA expression, which are more properly characterized under MGC models. It is also worth noting that a significant gene set identified by one of our methods could be driven by a small subset of very significant gene members (i.e., signals are sparse), whether the signal arises from the main effects of DNAm or RNA expression, or their interactions. Indeed, this is a distinct advantage of our approach, as sparse signals may nonetheless have high biological significance with respect to disease pathways (for example, in the case of CpG loci in gene promoter regions). In these cases, it is useful to conduct further locus-by-locus or gene-by-gene analyses characterizing the gene members in the set, as we did for our TCGA application and the Gautschi SRC signaling pathway.

## Conclusion

While the iNOTE approaches make fewer assumptions about the underlying causal disease models in a gene set, the tradeoff is an increase in computational burden. Both iNOTE methods are robust to model misspecification and, importantly, performed with close to optimal power across all simulations settings, particularly those in which the gene set is comprised of mixtures of different disease risk models – a highly likely biological scenario.

We propose two efficient procedures for gene set screening which use self-contained hypothesis tests, and therefore do not rely on the size or proportion of signals within, compared to without, the tested gene set. Furthermore, iNOTE and iTEGS can easily incorporate the adjustments for potential confounding covariates. Our methods dominated two competing methods with respect to power, and further recovered a much greater number of gene sets known a priori to be associated with lung cancer in our scans for gene sets associated with lung cancer survival. In particular, gene sets related to KRAS, EGFR, mir-let7a3 were found to be significantly associated with lung cancer survival. Finally, our methods are easily extended to include more or different genetic platforms. iTEGS and iNOTE software implemented in R in the present manuscript are available in Additional file 2. For any future updated versions, it may also be downloaded via the inote package at https://cran.r-project.org/.

## Additional files

Additional file 1 Supplementary information. Figure A Internal power simulation across various disease-model settings for moderately sized gene sets Figure B Power simulations comparing variance-component-based total effect gene set testing procedures to existing methods under mixture disease-model settings Table C : Davies approximation *p*-values for gene sets signficantly associated with lung cancer in TCGA subjects after Bonferroni correction Table D Counts of overlapping significant BIOCARTA/ KEGG gene sets associated with one-year lung cancer survival status by iTEGS, iNOTE, and GSAA Table E Counts of overlapping significant lung cancer gene sets associated with pathological stage of tumor at diagnosis by iTEGS, iNOTE, and GSAA; Table E.2: Variance component-based total effect test *p*-values for lung cancer gene sets significantly associated with pathological stage of tumor after Bonferroni correction. (PDF 2410 kb)

Additional file 2 iNote installable R package. (TAR 319 kb)

## Abbreviations

DNAm: DNA methylation
iTEGS: integrated total effect of a gene set
iNOTE: Integrated network omnibus total effect test
GSAA: Gene set association analysis
M: Risk model with only methylation effects
G: Risk model with only gene expression effects
MG: Risk model with only methylation and gene expression main effects
MGC: Risk model for methylation, gene expression, and their interactions
