# Supplementary material for: Integrated genomic analysis of biological gene sets with applications in lung cancer prognosis
Source: BMC Bioinformatics. 2017 Jul 11;18:336. doi: 10.1186/s12859-017-1737-2 (PMC5505153; doi:10.1186/s12859-017-1737-2)
Supplement: Supplementary file 1 — Supplementary information. Figure A Internal power simulation across various disease-model settings for moderately sized gene sets Figure B Power simulations comparing variance-component-based total effect gene set testing procedures to existing methods under mixture disease-model settings Table C : Davies approximation p-values for gene sets signficantly associated with lung cancer in TCGA subjects after Bonferroni correction Table D Counts of overlapping significant BIOCARTA/ KEGG gene sets associated with one-year lung cancer survival status by iTEGS, iNOTE, and GSAA Table E Counts of overlapping significant lung cancer gene sets associated with pathological stage of tumor at diagnosis by iTEGS, iNOTE, and GSAA; Table E.2: Variance component-based total effect test p-values for lung cancer gene sets significantly associated with pathological stage of tumor after Bonferroni correction. (PDF 2410 kb) [file 12859_2017_1737_MOESM1_ESM.pdf]

Supplement for "Integrated genomic analysis of biological gene sets with applications in lung cancer prognosis"

A Power comparisons of iNOTE and iTEGS across additional simulation settings.

A.1 iNOTE and iTEGS power simulations across mixture disease-model settings for moderately sized gene sets.

Power performance is shown in Figure A.1 for a gene set of size 50 with a 20% causal risk signal proportion of genes under the disease model settings where all causal genes contribute to disease via a) an equal mixture of M and MG; b) an equal mixture of M and MGC; c) an equal mixture of MG and MGC. κ on the x-axis denotes the coefficient multiplier for each of the effects β_M, β_{MG}, and β_{MGC}.

As observed in the simulations reported in the main text for an equal mixture of M, MG and MGC causal risk genes, iTEGS-M performs poorly in settings where both methylation and gene expression effects are present, while iTEGS-MG demonstrates the best performance across all three additional mixture simulations. The iTEGS-MGC and iNOTE methods perform nearly as well as the iTEGS-MG.

A.2 iNOTE and iTEGS power simulations for moderately sized gene sets under high causal signal density proportion.

Power performance is shown in Figure A.2 for a gene set of size 50 with an 80% causal risk signal proportion of genes under the underlying disease risk model settings where all causal genes contribute to disease risk via a) methylation effect only (M); b) methylation and mRNA expression effect (MG); c) methylation, MRNA expression, and their interactive effects (MGC); d) an equal mixture of M and MG; e) an equal mixture of M and MGC; f) an equal mixture of MG and MGC; g) an equal mixture of M, MG, and MGC. κ on the x-axis denotes the coefficient multiplier for each of the effects β_M, β_{MG}, and β_{MGC}.

These simulations increase the number of causal genes in the gene set of size 50 from 10 genes to 40. This increase in the causal risk signal proportion results in a much earlier, and rapid increase in power performance with respect to the coefficient multiplier, κ. In other words, the power is very high even when the individual causal risk associations for each gene is low.

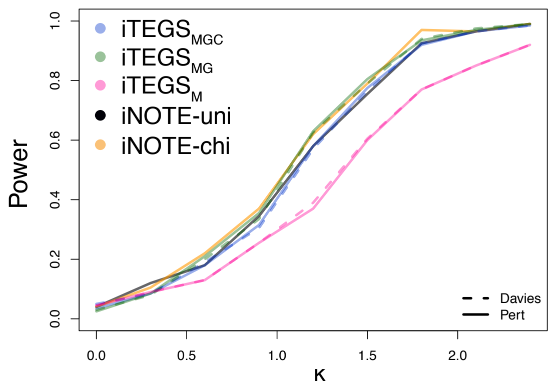

(a) M:MG: $\beta_M = 0.05, \beta_{MG} = 0.05; \beta_{MGC} = 0$

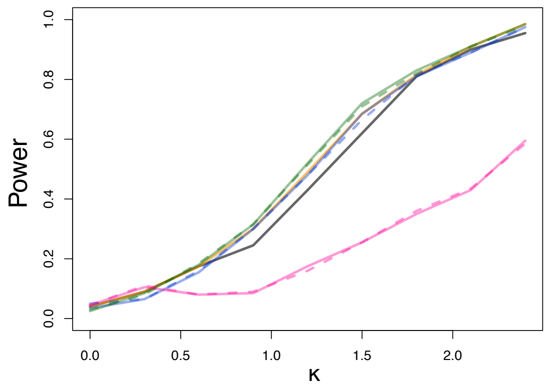

(b) M:MGC: $\beta_M = 0.05, \beta_{MG} = 0.05; \beta_{MGC} = 0.05$

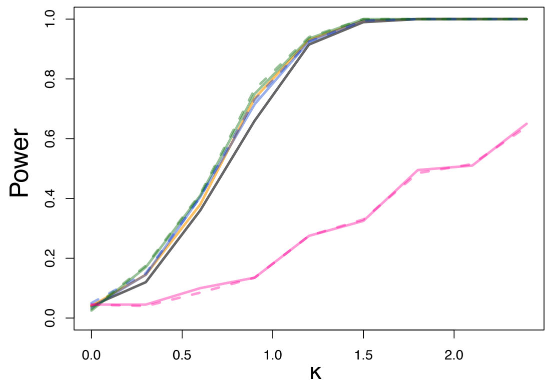

(c) MG:MGC: $\beta_M = 0.05, \beta_{MG} = 0.05; \beta_{MGC} = 0.05$

Figure A.1: Internal power simulation across various disease-model settings for moderately sized gene sets, with signal density proportion 20%

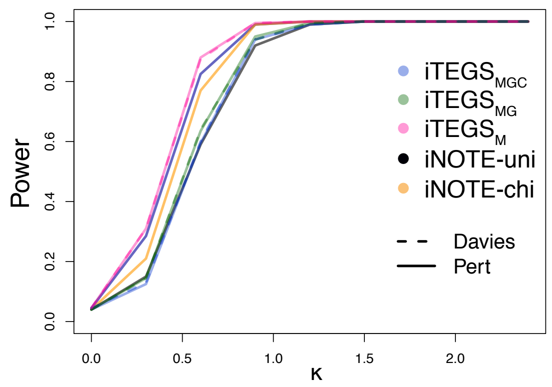

(a) M: $\beta_M = 0.05, \beta_{MG} = 0; \beta_{MGC} = 0$

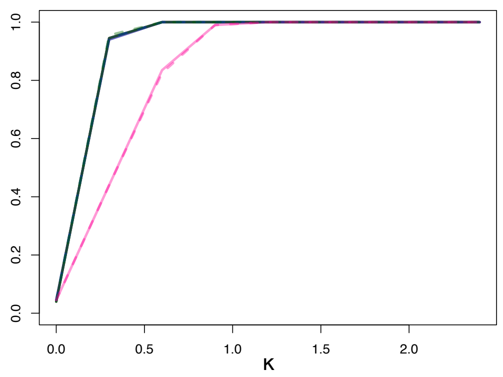

(b) MG: $\beta_M = 0.05, \beta_{MG} = 0.05; \beta_{MGC} = 0$

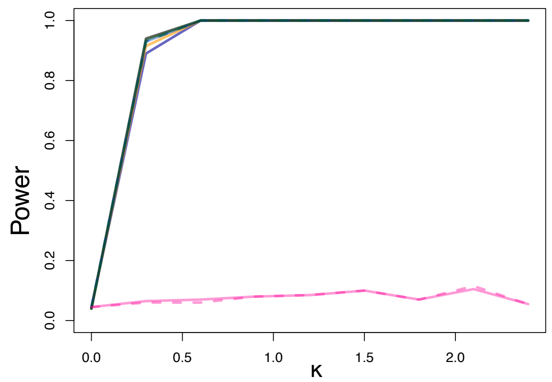

(c) MGC: $\beta_M = 0.05, \beta_{MG} = 0.05; \beta_{MGC} = 0.05$

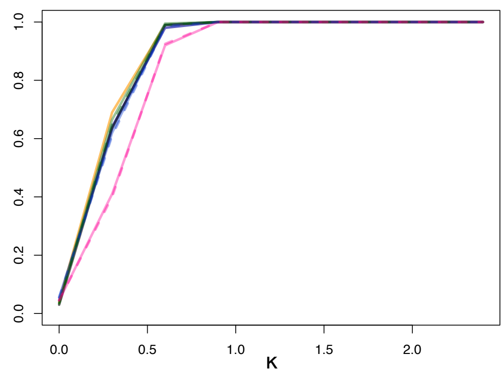

(d) M:MG: $\beta_M = 0.05, \beta_{MG} = 0.05; \beta_{MGC} = 0$

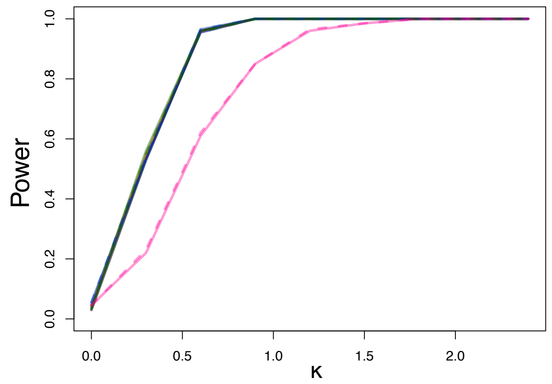

(e) M:MGC: $\beta_M = 0.05, \beta_{MG} = 0.05; \beta_{MGC} = 0.05$

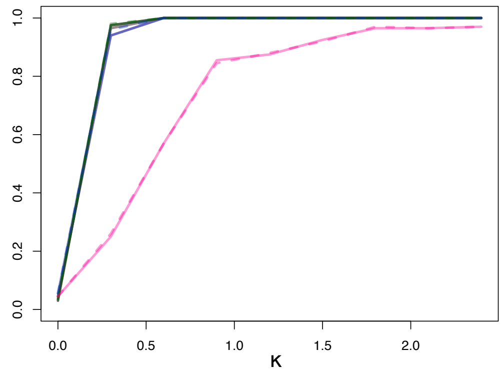

(f) MG:MGC: $\beta_M = 0.05, \beta_{MG} = 0.05; \beta_{MGC} = 0.05$

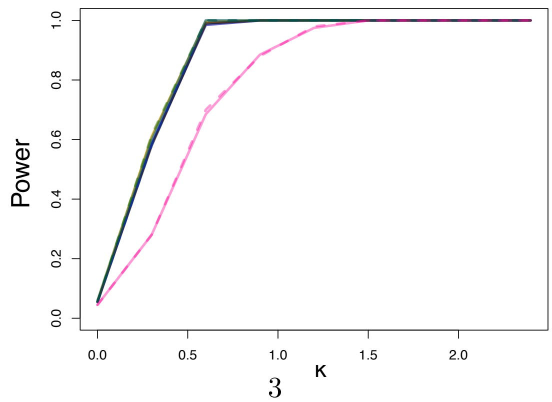

(g) M:MG:MGC: $\beta_M = 0.05, \beta_{MG} = 0.05; \beta_{MGC} = 0.05$

Figure A.2: Internal power simulation across various disease-model settings for moderately sized gene sets, with signal density proportion 80%

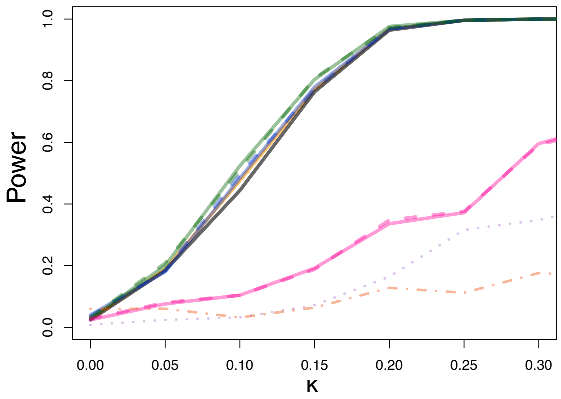

(a) M:MG: $\beta_M = 0.05, \beta_{MG} = 0.05; \beta_{MGC} = 0$

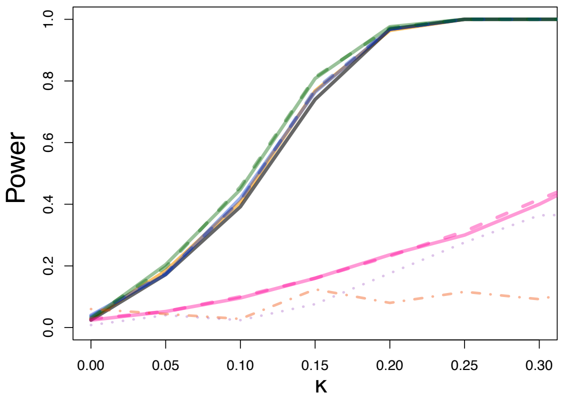

(b) M:MGC: $\beta_M = 0.05, \beta_{MG} = 0.05; \beta_{MGC} = 0.05$

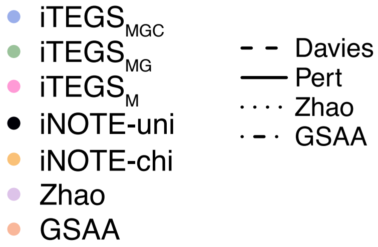

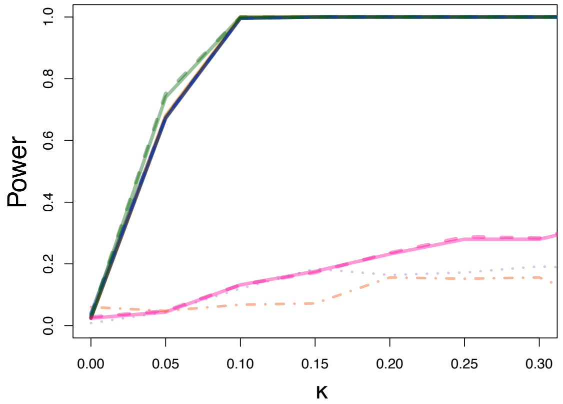

(c) MG:MGC: $\beta_M = 0.05, \beta_{MG} = 0.05; \beta_{MGC} = 0.05$

Figure B.1: Power simulations comparing variance-component-based total effect gene set testing procedures to existing methods under mixture disease-model settings.

B Power simulations comparing variance-component-based total effect gene set testing procedures to existing methods under mixture disease-model settings

Power performance is shown in Figure B.1 for a gene set of size 3 with a 100% causal risk signal proportion of genes under the underlying disease risk model settings where all causal genes contribute to disease risk via a) an equal mixture of M and MG; b) an equal mixture of M and MGC; c) an equal mixture of MG and MGC.

In these additional mixture simulations, the power results for each method are similar to those presented in the main text. The iNOTE and iTEGS methods, including the poor-performing iTEGS-M for mixture-disease models, consistently outperform the Zhao and GSAA methods.

Table C.1: Davies approximation p-values for gene sets signficantly associated with lung cancer in TCGA subjects after Bonferroni correction.

			Davies Approximation P-Values			
	N_0	N_T	Q_{MGC}	Q_{MG}	Q_M	Q_G
BRUECKNER TARGETS OF MIRLET7A3 DN	78	71	0.08	0.097	0.246	0.03
BRUECKNER TARGETS OF MIRLET7A3 UP	111	106	0.107	0.14	0.285	0.046
COLDREN GEFITINIB RESISTANCE DN	230	216	0.222	0.216	0.232	0.231
DAUER STAT3 TARGETS UP	49	49	0.055	0.079	0.246	0.029
DCA UP.V1 DN	193	163	0.244	0.269	0.385	0.115
DCA UP.V1 UP	191	162	0.242	0.261	0.346	0.142
HALMOS CEBPA TARGETS DN	46	44	0.098	0.134	0.321	0.035
KIM MYC AMPLIFICATION TARGETS UP	201	169	0.197	0.242	0.416	0.087
HATADA METHYLATED IN LUNG CANCER UP	390	356	0.286	0.314	0.364	0.224
KOBAYASHI EGFR SIGNALING 24HR DN	251	228	0.333	0.323	0.303	0.319
KOBAYASHI EGFR SIGNALING 24HR UP	101	91	0.108	0.15	0.322	0.048
KOBAYASHI EGFR SIGNALING 6HR DN	18	18	0.013	0.024	0.129	0.015
KRAS.600.LUNG.BREAST UP.V1 DN	289	261	0.345	0.379	0.494	0.169
KRAS.600.LUNG.BREAST UP.V1 UP	288	247	0.203	0.268	0.521	0.037
KRAS.AMP.LUNG UP.V1 UP	144	121	0.237	0.275	0.532	0.038
KRAS.DF.V1 UP	193	175	0.088	0.14	0.347	0.021
KRAS.LUNG.BREAST UP.V1 UP	145	127	0.193	0.276	0.575	0.038
KRAS.LUNG UP.V1 UP	141	126	0.065	0.121	0.432	0.009
LI AMPLIFIED IN LUNG CANCER	178	165	0.269	0.28	0.293	0.284
LOCKWOOD AMPLIFIED IN LUNG CANCER	214	205	0.341	0.316	0.285	0.418
MAYBURD RESPONSE TO L663536 UP	29	23	0.059	0.109	0.353	0.019
SHEDDEN LUNG CANCER GOOD SURVIVAL A12	317	269	0.204	0.258	0.421	0.074
SHEDDEN LUNG CANCER GOOD SURVIVAL A4	196	186	0.303	0.324	0.344	0.294
SHEDDEN LUNG CANCER POOR SURVIVAL A6	456	411	0.273	0.263	0.277	0.247
SWEET KRAS ONCOGENIC SIGNATURE	89	81	0.281	0.226	0.203	0.326
SWEET KRAS TARGETS DN	66	59	0.024	0.038	0.225	0.002
TBK1.DF DN	287	266	0.214	0.242	0.341	0.122
TBK1.DF UP	290	275	0.422	0.414	0.397	0.449
TOOKER GEMCITABINE RESISTANCE DN	122	115	0.196	0.223	0.292	0.153
ZHONG RESPONSE TO AZACITIDINE AND TSA UP	183	158	0.231	0.243	0.26	0.248

Davies approximation p-values for for all significant gene sets after Bonferroni correction. N_0: total no. of genes in the gene set; N_T: total no. of genes with methylation and gene expression data available (i.e. tested); Q: the iTEGS Q-statistic test specifying M, G, MG, or MGC; Bonferroni adjusted p-value threshold was calculated as $\alpha/M = 5E-04$, where $\alpha = 0.05$ and M is the total number of gene sets tested.

C Variance component-based total effect test p-values for gene sets associated with lung cancer in TCGA subjects using the Davies approximation.

P-values calculated for the iTEGS tests using the Davies approximation are reported in Table C.1. As noted in the main text, the Davies approximations for iTEGS yield results that are similar to the empirical perturbation based iTEGS primarily when the gene set is small.

D Extensions to disease model selection panel in iTEGS and iNOTE, with additional applications in KEGG and BIOCARTA pathways.

As gene-expression only models are also a commonly assumed disease-risk model in literature, and indeed may exhibit greater power when signal from DNAm sites is sparse, we extended the model search of both iNOTE algorithms, chi and uni, to also consider the gene-expression only model, denoted by Q_G.

We used iTEGS, the extended disease-risk model panels for iNOTE, and GSAA to conduct exploratory scans of pathway databases (which included gene sets not necessarily specific to lung cancer) to see what gene sets could be recovered. Comparisons of total and overlapping counts of significant gene sets identified by each method are reported in Tables D.1 and D.2. The p-values for each gene set test under the iTEGS, iNOTE and GSAA methods are also reported for the top gene sets surviving Bonferroni correction for at least one iTEGS and at least one iNOTE test in Table D.3.

E Lung cancer MsigDB gene sets associated with pathological stage of tumor in TCGA subjects.

We conducted additional exploratory analyses in the MsigDB gene sets with known associations with lung cancer. In our additional analyses, we screened for gene sets associated with pathological stage of tumor at initial biopsy. Counts of total and overlapping gene sets identified by each method, iTEGS, iNOTE, and GSAA, are reported in Table E.1 and results for significant gene sets surviving Bonferroni correction in at least one iTEGS and at least one iNOTE test are reported in Table E.2.

Table D.1: Counts of overlapping significant BIOCARTA gene sets associated with one-year lung cancer survival status by iTEGS, iNOTE, and GSAA.

		iTEGS				iNOTE		GSAA
		MGC	MG	M	G	chi	uni	
iTEGS	MGC	32 (3)	31 (2)	19 (0)	16 (2)	26 (2)	25 (2)	1 (0)
	MG		43 (3)	31 (0)	15 (2)	31 (3)	33 (2)	1 (0)
	M			66 (0)	5 (0)	24 (0)	36 (0)	1 (0)
	G				17 (2)	12 (2)	14 (2)	0
iNOTE	chi					35 (6)	26 (2)	1 (0)
	uni						45 (2)	1 (0)
GSAA								5 (0)

A total of 214 BIOCARTA gene sets were obtained and tested from MsigDB.Tests for iTEGS were calculated under disease-risk model specifications M: methylation effect only, G: mRNA expression effect only, MG: methyation and mRNA expression effects, and MGC: methylation effect, mRNA expression effect, and their interactions. The total and overlapping counts of significant gene sets identified by each method is reported here, with numbers in parentheses denoting the counts of gene sets that remain significant after Bonferroni correction.

Table D.2: Counts of overlapping significant KEGG gene sets associated with one-year lung cancer survival status by iTEGS, iNOTE, and GSAA.

		iTEGS				iNOTE		GSAA
		MGC	MG	M	G	chi	uni	
iTEGS	MGC	98 (24)	94 (23)	60 (2)	63 (14)	83 (23)	85 (19)	3 (0)
	MG		115 (28)	79 (5)	63 (14)	86 (26)	99 (21)	3 (0)
	M			93 (9)	32 (1)	57 (3)	80 (6)	2 (0)
	G				64 (16)	59 (16)	60 (15)	2 (0)
iNOTE	chi					91 (41)	82 (22)	2 (0)
	uni						108 (25)	2 (0)
GSAA								5 (0)

A total of 175 KEGG gene sets were obtained and tested from MsigDB. Tests for iTEGS were calculated under disease-risk model specifications M: methylation effect only, G: mRNA expression effect only, MG: methyation and mRNA expression effects, and MGC: methylation effect, mRNA expression effect, and their interactions. The total and overlapping counts of significant gene sets identified by each method is reported here, with numbers in parentheses denoting the counts of gene sets that remain significant after Bonferroni correction.

Table D.3: Counts of overlapping significant KEGG gene sets associated with one-year lung cancer survival status by iTEGS, iNOTE, and GSAA.

	N_0	N_T	Q_{MGC}	Q_{MG}	Q_M	Q_G	$iNOTE_{chi}$	$iNOTE_{uni}$	GSAA
BIOCARTA CELL2CELL PATHWAY	14	14	5.39E-06	1.67E-05	0.420	3.07E-06	<1E-04	<1E-04	0.212
BIOCARTA EIF2 PATHWAY	11	10	5.27E-04	1.63E-04	0.004	0.005	1E-04	5E-04	0.900
BIOCARTA PPARA PATHWAY	58	55	1.11E-10	1.47E-10	0.010	1.60E-08	<1E-04	<1E-04	0.916
KEGG ALDOSTERONE REGULATED SODIUM REABSORPTION	42	39	9.01E-05	2.26E-04	0.050	7.84E-04	<1E-04	9E-04	0.008
KEGG ARACHIDONIC ACID METABOLISM	58	56	2.17E-04	2.60E-04	0.225	1.03E-04	<1E-04	4E-04	0.702
KEGG ARGININE AND PROLINE METABOLISM	54	46	2.08E-06	2.87E-06	0.004	1.01E-04	<1E-04	<1E-04	0.170
KEGG ARRHYTHMOGENIC RIGHT VENTRICULAR CARDIOMYOPATHY ARVC	76	71	2.71E-05	1.21E-05	0.011	2.49E-04	1E-04	<1E-04	0.892
KEGG BUTANOATE METABOLISM	34	32	2.46E-05	1.10E-04	0.006	0.002	<1E-04	<1E-04	0.696
KEGG ENDOCYTOSIS	183	173	0.027	1.18E-03	2.52E-07	0.807	0.009	<1E-04	0.722
KEGG ETHER LIPID METABOLISM	33	31	7.68E-05	1.37E-05	0.058	3.70E-05	<1E-04	<1E-04	0.478
KEGG FATTY ACID METABOLISM	42	38	9.14E-06	2.30E-05	0.003	8.20E-04	2E-04	<1E-04	0.968
KEGG GLUTATHIONE METABOLISM	50	46	1.30E-03	2.55E-04	0.002	0.010	<1E-04	8E-04	0.246
KEGG GLYCEROLIPID METABOLISM	49	46	6.92E-05	8.64E-05	0.053	1.81E-04	<1E-04	<1E-04	0.428
KEGG GLYCEROPHOSPHOLIPID METABOLISM	77	71	1.08E-07	1.82E-08	6.88E-04	5.55E-06	<1E-04	<1E-04	0.302
KEGG GLYCOLYSIS GLUCONEOGENESIS	62	58	6.73E-06	8.69E-05	0.020	5.70E-04	<1E-04	<1E-04	0.112
KEGG INSULIN SIGNALING PATHWAY	137	130	9.80E-09	1.12E-09	1.71E-06	4.80E-05	<1E-04	<1E-04	0.744
KEGG LEUKOCYTE TRANSENDOTHELIAL MIGRATION	118	109	7.56E-05	1.71E-04	0.028	5.55E-04	<1E-04	2E-04	0.530
KEGG LINOLEIC ACID METABOLISM	29	27	0.002	6.65E-04	0.603	6.92E-05	<1E-04	<1E-04	0.232
KEGG LONG TERM DEPRESSION	70	64	9.53E-07	1.76E-05	0.392	2.06E-06	<1E-04	<1E-04	0.632
KEGG LYSOSOME	121	113	0.006	6.75E-04	2.32E-04	0.139	0.014	2E-04	0.174
KEGG MAPK SIGNALING PATHWAY	267	249	1.12E-06	1.72E-08	2.14E-07	0.002	<1E-04	<1E-04	0.986
KEGG MATURITY ONSET DIABETES OF THE YOUNG	25	23	1.03E-07	4.83E-07	0.189	2.78E-07	<1E-04	<1E-04	0.192
KEGG OLFACTORY TRANSDUCTION	389	320	0.007	0.028	0.957	1.16E-05	<1E-04	<1E-04	0.902
KEGG PATHWAYS IN CANCER	328	311	0.003	1.72E-04	3.26E-05	0.104	0.029	<1E-04	0.994
KEGG PPAR SIGNALING PATHWAY	69	65	7.37E-10	4.05E-09	0.523	2.20E-10	<1E-04	<1E-04	0.450
KEGG PYRUVATE METABOLISM	40	37	2.58E-05	1.59E-04	0.011	0.002	<1E-04	5E-04	0.362
KEGG REGULATION OF ACTIN CYTOSKELETON	216	196	4.21E-06	5.59E-06	0.020	2.50E-05	<1E-04	<1E-04	0.928
KEGG STARCH AND SUCROSE METABOLISM	52	35	1.64E-06	4.40E-06	0.045	1.77E-05	<1E-04	<1E-04	0.338
KEGG TIGHT JUNCTION	134	128	1.68E-06	7.85E-06	0.024	2.61E-05	<1E-04	<1E-04	0.552
KEGG TRYPTOPHAN METABOLISM	40	37	5.73E-05	3.96E-05	0.007	6.45E-04	<1E-04	<1E-04	0.186
KEGG VALINE LEUCINE AND ISOLEUCINE DEGRADATION	44	41	7.61E-06	4.72E-05	0.017	2.10E-04	1E-04	<1E-04	0.390
KEGG VASOPRESSIN REGULATED WATER REABSORPTION	44	40	1.06E-04	9.13E-05	0.002	0.005	<1E-04	6E-04	0.148
KEGG VEGF SIGNALING PATHWAY	76	73	4.32E-04	6.03E-05	0.024	3.43E-04	<1E-04	4E-04	0.770
KEGG WNT SIGNALING PATHWAY	151	145	3.50E-04	5.55E-05	9.24E-05	0.020	1E-04	<1E-04	0.990

Satterthwaite approximated p-values for all significant gene sets surviving Bonferroni correction in at least one iTEGS and one iNOTE approach. N_0: total no. of genes in the gene set; N_T: total no. of genes with methylation and gene expression data available (i.e. tested); Q: the iTEGS Q-statistic test specifying M, G, MG, or MGC; Bonferroni adjusted p-value threshold was calculated as $\alpha/M = 5E-04$, where $\alpha = 0.05$ and M is the total number of gene sets tested. Model selection by the omnibus testing procedures include the Q_G in consideration.

Table E.1: Counts of overlapping significant lung cancer gene sets associated with pathological stage of tumor at diagnosis by iTEGS, iNOTE, and GSAA.

		iTEGS				iNOTE		GSAA
		MGC	MG	M	G	chi	uni	
iTEGS	MGC	68 (32)	67 (28)	33 (8)	58 (24)	63 (30)	61 (30)	3 (0)
	MG		72 (31)	37 (10)	59 (22)	65 (31)	62 (29)	3 (0)
	M			38 (10)	27 (5)	32 (10)	31(10)	2 (0)
	G				62 (24)	60 (24)	58 (24)	3 (0)
iNOTE	chi					68 (35)	61(31)	3 (0)
	uni						62 (32)	3 (0)
GSAA								4 (0)

A total of 99 lung cancer associated gene sets were obtained and tested from MsigDB. Tests for iTEGS were calculated under disease-risk model specifications M: methylation effect only, G: mRNA expression effect only, MG: methyation and mRNA expression effects, and MGC: methylation effect, mRNA expression effect, and their interactions.The total and overlapping counts of significant gene sets identified by each method is reported here, with numbers in parentheses denoting the counts of gene sets that remain significant after Bonferroni correction.

Table E.2: Variance component-based total effect test p-values for lung cancer gene sets significantly associated with pathological stage of tumor after Bonferroni correction.

			Approximated P-Values				Empirical P-Values				Omnibus P-Values		GSAA
	N_0	N_T	Q_{MGC}	Q_{MG}	Q_M	Q_G	Q_{MGC}	Q_{MG}	Q_M	Q_G	$iNOTE_{chi}$	$iNOTE_{uni}$	GSAA
BRUECKNER TARGETS OF MIRLET7A3 UP	111	106	8.09E-12	1.96E-11	3.03E-05	1.02E-07	<1E-04	<1E-04	<1E-04	<1E-04	<1E-04	<1E-04	1.000
COLDREN GEFITINIB RESISTANCE DN	230	216	0.001	8.77E-05	1.07E-04	0.036	0.001	1E-04	2E-04	0.035	<1E-04	<1E-04	0.774
COLDREN GEFITINIB RESISTANCE UP	85	75	4.39E-10	1.61E-08	0.176	7.02E-09	<1E-04	<1E-04	0.175	<1E-04	<1E-04	<1E-04	1.000
DCA UP.V1 DN	193	163	5.04E-07	2.58E-06	0.326	3.63E-08	<1E-04	<1E-04	0.322	<1E-04	<1E-04	<1E-04	0.070
DCA UP.V1 UP	191	162	5.14E-07	4.19E-06	0.033	9.60E-06	<1E-04	<1E-04	0.035	1E-04	<1E-04	<1E-04	0.040
HATADA METHYLATED IN LUNG CANCER UP	390	356	1.86E-10	4.96E-08	0.007	3.37E-07	<1E-04	<1E-04	0.008	<1E-04	<1E-04	<1E-04	0.508
JEON SMAD6 TARGETS DN	19	18	1.78E-05	4.73E-05	0.007	0.001	<1E-04	2E-04	0.010	0.001	1E-04	<1E-04	0.912
KEGG NON SMALL CELL LUNG CANCER	54	53	5.43E-05	2.21E-04	0.111	1.61E-04	1E-04	6E-04	0.109	1E-04	<1E-04	<1E-04	0.824
KEGG SMALL CELL LUNG CANCER	84	79	9.99E-06	1.66E-05	0.011	1.79E-04	1E-04	1E-04	0.013	4E-04	1E-04	<1E-04	0.938
KIM MYCN AMPLIFICATION TARGETS UP	92	81	3.21E-05	7.14E-05	0.057	1.11E-04	<1E-04	2E-04	0.058	1E-04	1E-04	<1E-04	0.634
KOBAYASHI EGFR SIGNALING 24HR DN	251	228	5.14E-17	1.42E-12	0.125	3.23E-15	<1E-04	<1E-04	0.123	<1E-04	<1E-04	<1E-04	0.958
KOBAYASHI EGFR SIGNALING 24HR UP	101	91	4.57E-06	1.93E-05	0.036	6.80E-05	<1E-04	<1E-04	0.036	<1E-04	<1E-04	<1E-04	0.116
KRAS.600.LUNG.BREAST UP.V1 DN	289	261	2.06E-07	5.84E-06	0.090	3.05E-06	<1E-04	<1E-04	0.091	<1E-04	<1E-04	<1E-04	0.054
KRAS.600.LUNG.BREAST UP.V1 UP	288	247	3.80E-05	2.65E-04	0.391	1.28E-05	3E-04	9E-04	0.391	1E-04	<1E-04	<1E-04	0.368
KRAS.AMP.LUNG UP.V1 DN	146	127	1.29E-04	6.42E-04	0.725	4.87E-06	2E-04	0.002	0.724	<1E-04	<1E-04	<1E-04	0.096
KRAS.AMP.LUNG UP.V1 UP	144	121	3.16E-05	4.16E-04	0.309	4.71E-05	2E-04	4E-04	0.304	<1E-04	<1E-04	<1E-04	0.106
KRAS.DF.V1 DN	194	177	3.77E-04	2.24E-04	0.031	0.001	5E-04	4E-04	0.034	0.002	1E-04	0.001	0.608
KRAS.DF.V1 UP	193	175	1.77E-06	2.48E-06	3.29E-04	7.49E-04	<1E-04	<1E-04	4E-04	6E-04	<1E-04	<1E-04	0.940
KRAS.LUNG.BREAST UP.V1 UP	145	127	1.39E-05	4.07E-05	0.217	5.12E-06	<1E-04	1E-04	0.215	<1E-04	<1E-04	<1E-04	0.216
KRAS.LUNG UP.V1 UP	141	126	1.27E-04	8.63E-04	0.408	6.67E-05	<1E-04	5E-04	0.406	<1E-04	<1E-04	<1E-04	0.112
LOCKWOOD AMPLIFIED IN LUNG CANCER	214	205	3.87E-04	9.46E-05	6.12E-04	0.015	7E-04	1E-04	9E-04	0.0146	<1E-04	<1E-04	0.846
OSADA ASCL1 TARGETS DN	24	23	1.13E-05	1.59E-05	3.39E-04	0.003	1E-04	<1E-04	5E-04	0.0037	<1E-04	<1E-04	0.926
SHEDDEN LUNG CANCER GOOD SURVIVAL A12	317	269	4.59E-12	2.00E-11	0.001	1.06E-09	<1E-04	<1E-04	0.001	<1E-04	<1E-04	<1E-04	0.080
SHEDDEN LUNG CANCER GOOD SURVIVAL A4	196	186	1.01E-11	1.68E-13	2.61E-08	3.09E-07	<1E-04	<1E-04	<1E-04	<1E-04	<1E-04	<1E-04	0.292
SHEDDEN LUNG CANCER POOR SURVIVAL A6	456	411	3.43E-25	5.51E-20	0.020	1.02E-22	<1E-04	<1E-04	0.021	<1E-04	<1E-04	<1E-04	0.986
SWEET KRAS ONCOGENIC SIGNATURE	89	81	4.69E-05	9.66E-06	0.004	3.74E-04	1E-04	<1E-04	0.004	2E-04	<1E-04	<1E-04	0.834
SWEET KRAS TARGETS DN	66	59	8.89E-05	0.001	0.051	0.003	2E-04	0.002	0.054	0.005	0.001	3E-04	0.910
SWEET KRAS TARGETS UP	84	80	1.16E-05	6.64E-06	3.35E-04	0.002	<1E-04	<1E-04	4E-04	0.001	<1E-04	<1E-04	0.746
TBK1.DF DN	287	266	5.87E-13	1.68E-13	1.26E-07	1.23E-07	<1E-04	<1E-04	<1E-04	<1E-04	<1E-04	<1E-04	0.986
TBK1.DF UP	290	275	1.65E-06	9.30E-07	1.67E-04	3.90E-04	<1E-04	<1E-04	3E-04	4E-04	<1E-04	<1E-04	0.672
TOMIDA METASTASIS DN	18	16	8.03E-04	3.69E-04	5.15E-05	0.130	0.002	7E-04	1E-04	0.128	1E-04	<1E-04	0.864
TOOKER GEMCITABINE RESISTANCE DN	122	115	2.85E-08	3.62E-08	2.26E-04	3.40E-05	<1E-04	<1E-04	3E-04	<1E-04	<1E-04	<1E-04	0.998
ZHONG RESPONSE TO AZACITIDINE AND TSA UP	183	158	9.44E-10	3.65E-08	0.031	6.34E-08	<1E-04	<1E-04	0.032	<1E-04	<1E-04	<1E-04	0.910

Satterthwaite approximated and empirical p-values for network tests for all significant gene sets after Bonferroni correction. Empirical p-values and approximated p-values are nearly equivalent, irrespective of the sizes of the gene sets tested. N_0: total no. of genes in the gene set; N_T: total no. of genes with methylation and gene expression data available (i.e. tested); Q: the iTEGS Q-statistic test specifying M, G, MG, or MGC; Bonferroni adjusted p-value threshold was calculated as $\alpha/M = 5E-04$, where $\alpha = 0.05$ and M is the total number of gene sets tested. Model selection by the omnibus testing procedures include the Q_G in consideration.
